# Pharmacokinetics and Pharmacodynamics of the Combination of Rhein and Curcumin in the Treatment of Chronic Kidney Disease in Rats

**DOI:** 10.3389/fphar.2020.573118

**Published:** 2020-12-23

**Authors:** Xiaoying He, Guowei Li, Yuanyuan Chen, Qiming Xiao, Xinwei Yu, Xixi Yu, Xiaoyang Lu, Zheng Xiang

**Affiliations:** ^1^ School of Pharmaceutical Sciences, Wenzhou Medical University, Wenzhou, China; ^2^ Zhejiang Provincial Key Laboratory for Drug Evaluation and Clinical Research, The First Affiliated Hospital, Zhejiang University, Zhejiang, China

**Keywords:** pharmacodynamics, Pharmacokinetics, chronic kidney disease, molecular docking, curcumin, rhein

## Abstract

**Objectives:** The interaction between the components of traditional Chinese medicine (TCM) is an important basis for their synergy. Rhein and curcumin exert various pharmacological activities, including anti-tumour, anti-inflammatory, antioxidant, anti-fibrosis and renoprotective effects. However, no investigation has reported the synergistic anti-fibrosis effect yet. This study aims at determine the pharmacokinetics and pharmacodynamics of the combination of rhein and curcumin in the treatment for chronic kidney disease in rats.

**Design:** Fifty two male Sprague-Dawley (SD) rats were randomly divided into rhein group, curcumin group and their combination group for pharmacodynamics studies. HE and Masson staining was conducted to observe the changes of renal morphology. Kits were used to detect the level of urea nitrogen (BUN) and creatinine (Scr). For pharmacokinetic study, 36 SD rats were randomly divided into rhein group, curcumin group and a combination group, the content of rhein and curcumin in plasma and renal tissue was determined by ultra-performance liquid chromatography-tandem mass spectrometry (UPLC-MS/MS). In additon, molecular docking method and cell experiments was used to disclose the interaction mechanism between curcumin and rhein.

**Results:** The pharmacodynamic results showed that the degree of renal fibrosis was improved obviously by co-administration rhein and curcumin. Meanwhile, compared to single administration, the Cmax and AUC of rhein and curcumin in plasma and renal tissue were enhanced significantly after co-administration. Moreover, the result of molecular docking and cell experiments showed that both two compounds could interact with P-gp, CYP2C9 and CYP2C19.

**Conclusion:** Together, these findings demonstrated that rhein and curcumin had a synergistic effect in ameliorateing chonic kidney disease, providing an important explanation on the synergistic mechanism of curcumin and rhein from a pharmacokinetic viewpoint.

## Introduction

According to epidemiological survey, the total prevalence of chronic kidney disease (CKD) in the world is 11% (Gansevoort et al., 2013; Xie et al., 2018). However, there are no drugs for the treatment of renal fibrosis in the clinic at present. Traditional Chinese medicine (TCM) has a long history and unique curative effect on the treatment of CKD (Chen et al., 2019a). The previous study showed that TCM including *Uremic Clearance granule*, *Dahuang huichong pill, Kangxianling formula* and *Shenkang* Injection have unique efficacy in the treatment of CKD (Cao et al., 2017). Rhein, derived from *Rheum palmatum* L. (Li et al., 2015), is considered as the major active ingredient of these Chinese medicinal formulas. It possesses various pharmacological activities such as anti-tumour, anti-inflammatory, antibacterial, antioxidant, antifibrosis and renoprotective activity (Martin et al., 2003; Wang et al., 2012). Curcumin, mainly derived from dried rhizome of *Curcuma longa L*, possesses multiple pharmacological abilities, including anticancer, anti-inflammatory, antioxidation, antifibrotic, anti-arthritis (Hatcher et al., 2008; Azhdari et al., 2019), as well as a neuroprotective effect (Sun et al., 2017). Both rhein and curcumin have antifibrotic activity and have showed good efficacy to prevent the progress of renal fibrosis (Kuwabara et al., 2006; He et al., 2011; Soetikno et al., 2013; Zhou et al., 2014; Ho et al., 2016; Hu et al., 2016; Zhang et al., 2016; Chen et al., 2019b). Therefore, further investigation of the antifibrosis effect of rhein and curcumin was of great significance for the development of new antifibrosis drugs.

Several studies have been reported on the herb-drug interactions of rhein and curcumin. It was demonstrated that curcumin could change the pharmacokinetic profile of peroral celiprolol, losartan, loratadine tamoxifen (Bahramsoltani et al., 2017). Also, rhein showed a strong synergistic effect with known drugs such as doxorubicin (Tang et al., 2009), methotrexate (Yuan et al., 2017). Our previous studies demonstrated that Bu-shen-Huo-xue formula (BSHX) could effectively alleviate renal interstitial fibrosis (Shi et al., 2014; Xiang et al., 2015; Sun et al., 2016). Rhein and curcumin were considered as the main effective ingredients of BSHX (Shi et al., 2014). Therefore, it is necessary to study the efficacy and mechanism of their coadministration. In this study, we focused on the pharmacokinetics and pharmacodynamics of co-administration of rhein and curcumin for the treatment of CKD in rats, and examined the synergistic compatibility between these compounds from a pharmacokinetic viewpoint.

## Methods

### Chemical and Reagents

Chemical reference substances with purity >98%, including rhein, curcumin and neosperidin dihydrochalcone (NHDC), were purchased by Chengdu Mansite (Sichuan, China). HPLC grade formic acid, methanol and acetonitrile were provided from Merck (Merck, Darmstadt, Germany). The kits for Creatinine (Cre), urea nitrogen (BUN) were obtained by Nanjing Jiancheng Bioengineering Research Institute (Nanjing, China). Masson and HE staining solution kits were purchased by Beijing Solable Technology Co., Ltd (Beijing, China). Other reagents used in the experiment were all analytical purity.

### Animal Experiments

Sprague-Dawley (SD) rats (200 ± 20 g) were purchased from Laboratory Animal Center of Wenzhou Medical University (Wenzhou, China). The rats were kept in an air-conditioned house with the temperature of 24 ± 2°C, an appropriate humidity of 40–60% and a 12 h dark/light cycle. All animal had access to food and water during the experimental period and were adapted for one week. All animal care and experimental procedures were complied with the “Principles of Laboratory Animal Care” and approved by the Animal Ethics Committee in Wenzhou Medical University (July 25, 2018, no: WMU-2018–0024). The animal experiments were carried out according to the European Community guidelines for the use of experimental animals.

For the pharmacodynamics study, 52 SD rats were randomly divided into thirteen groups (n = 4 each group): sham group; Unilateral Ureteral Obstruct (UUO) model group (7^th^, 14th, 21st day); rhein treatment group (7^th^, 14th, 21st day); curcumin treatment group (7^th^, 14th, 21st day); co-administration of rhein and curcumin treatment group (7^th^, 14th, 21st day). For UUO operation, rats were deeply anesthetized using 10% chloral hydrate (0.3 ml/100 g body weight). A midline incision was made to expose the abdominal cavity, and the right ureter was separated for ligation. In sham-operated group, right ureter were only separated but not ligated. The rats were administrated with rhein (100 mg/kg), curcumin (100 mg/kg) or rhein (100 mg/kg) plus curcumin (100 mg/kg) every day after UUO operation, while the sham-operated and UUO model group were given the same volume of saline every day.

For the pharmacokinetic study, 36 SD rats were randomly divided into six groups (n = 6 per group), and were administrated with rhein (100 mg/kg), curcumin (100 mg/kg), or rhein (100 mg/kg) plus curcumin (100 mg/kg), via gavage. Before the pharmacokinetic experiments, all rats were placed into metabolic cages and fasted overnight. The plasma of 250 µL was obtained in tubes with 10 μL heparin from the rat tail vein at 0.167, 0.5, 1, 2, 3, 4, 6, 8, 12, and 24 h after gavage administration. Then, the collected blood samples were centrifuged at 4°C for 15 min at 4,000 rpm, and were stored at −20°C in refrigerator until further analysis. In addition, tissue collection was conducted at 0.5, 1 and 2 h after oral gavage, respectively. Colleced tissues were washed with normal saline, dried, and placed on filter paper, and weighted. Homogenate was prepared according to the proportion of tissue weight and normal saline volume (1:2) and stored at −80°C in refrigerator until further analysis. At the end of the experiment, all rats were sacrificed by cervical dislocation.

### Sample Preparation

For the pharmacodynamic study, rats were sacrificed after the 7^th^, 14th and 21st day after administration. Before sacrifice, plasma was collected from rats’ tail vein for detecting the content of creatinine and urea nitrogen. Then, rats were deeply anesthetized with 10% chloral hydrate (0.3 ml per 100 g body weight). The right kidney was taken out after cardiac perfusion and fixed in paraformaldehyde for subsequent pathomorphological examination.

For the pharmacokinetic study, 100 μL plasma sample was mixed with 10 μL IS, and 290 µL acetonitrile was added for protein sedimentation. The mixture was centrifuged at 12,000 *g* at 4°C for 10 min after vortex. Next, 2 μL of the supernatant was injected into UPLC-MS/MS system.

### Pharmacodynamic Study

The content of Cre and BUN was determined using kits. Collected renal tissue was fixed using neutral buffered formalin for 24 h, and then dehydrated through a graded ethanol series. The renal tissue was then embedded in paraffin and transverse sections were made on a microtome at 3 μm thickness in order to HE and Masson examination. Light microscope was used to observe pathological-morphological changes in the kidney tissue.

### Pharmacokinetic Study

According to the latest Food and Drug Administration (FDA) guidelines Food and Drug Administration (2018), the method validation was accomplished in term of selectivity, linearity, precision, accuracy, matrix effect, recovery rate and stability to prove the reliability and reproducibility of the established method. The detailed method were provided in Supplementary Material.

The pharmacokinetic parameters were calculated by Drug and Statistics (DAS 3.0) software (Shanghai, China). The following pharmacokinetic parameters were listed: area under the curve from time (AUC_0-t_); elimination half-life (T_1/2_); total clearance (Cl); maximal plasma concentration (C_max_); volume of distribution (V_d_); time to reach maximal plasma concentration (T_max_). All pharmacokinetic parameters were expressed as mean ± standard deviation (SD). The SPSS software version 18 (SPSS, Inc.) was used to perform statistical analysis. Nonparametric method was selected for comparisons of the pharmacokinetic difference among rhein, curcumin and their combination. *p*-value below 0.05 was considered indicative of statistical significance.

### Docking Studies

Rhein and curcumin were docked with P-gp, CYP2C9 or CYP2C19, respectively. At first, the crystal structure of P-gp, CYP2C9 and CYP2C19 were downloaded from the Protein Data Bank, and were pre-processed with PYMOL software to remove water molecules and heteroatoms, add hydrogen atoms and fix non-hydrogen atoms. Then, the ligand position in P-gp, CYP2C9 and CYP2C19 were used to define the active site. Finally, the prepared files of molecular coordinate and lattice parameter were imported into the AUTODOCK 4.2.6 docking program. The docking score between the natural ligand and P-gp, CYP2C9 and CYP2C19 proteins was used as the cut-off value in this protocol. If the docking score between curcumin or rhein, and P-gp, CYP2C9 or CYP2C19 was less than the corresponding cut-off value of natural ligands, and less than -5.0 kcaL/mol, they were considered to be effective docking.

### Interaction Between Rhein and Curcumin *in vitro*

Recombinant human cytochrome P450 enzymes (CYP2C9) were provided from Reid Ltd, Research Institute for Liver Diseases (Shanghai, China). To explore the interaction between rhein and curcumin, curcumin (20 μM) was chosen as substrates and rhein was used as the inhibition. Incubation mixtures were prepared in a total volume of 200 μL containing 0.25 mg/ml recombinant human cytochrome P450 enzymes, potassium phosphate buffer (pH 7.4), an NADPH generating system [5 mM glucose 6-phosphate, 1 mM NADP+, 1 U/mL glucose 6-phosphate dehydrogenase]. A series concentration of rhein (final concentrations were 1, 10, and 50 μM) were incubated with curcumin for 60 min at 37°C. The quality control samples were incubated without rhein. The reactions were stopped by adding 2-fold volume (200 μL) of cold acetonitrile. The assay was performed in triplicate for all test specimens. Then, the samples were vortexed and centrifuged at 13, 000 rpm for 10 min to precipitate protein. The supernatant was analyzed by UPLC- MS/MS, and the inhibitory effect of rhein was assessed by the difference in the residual amount curcumin between samples and the corresponding quality control samples.

## Results

### Pharmacodynamic Study

#### HE and Masson Staining

**FIGURE 1:**
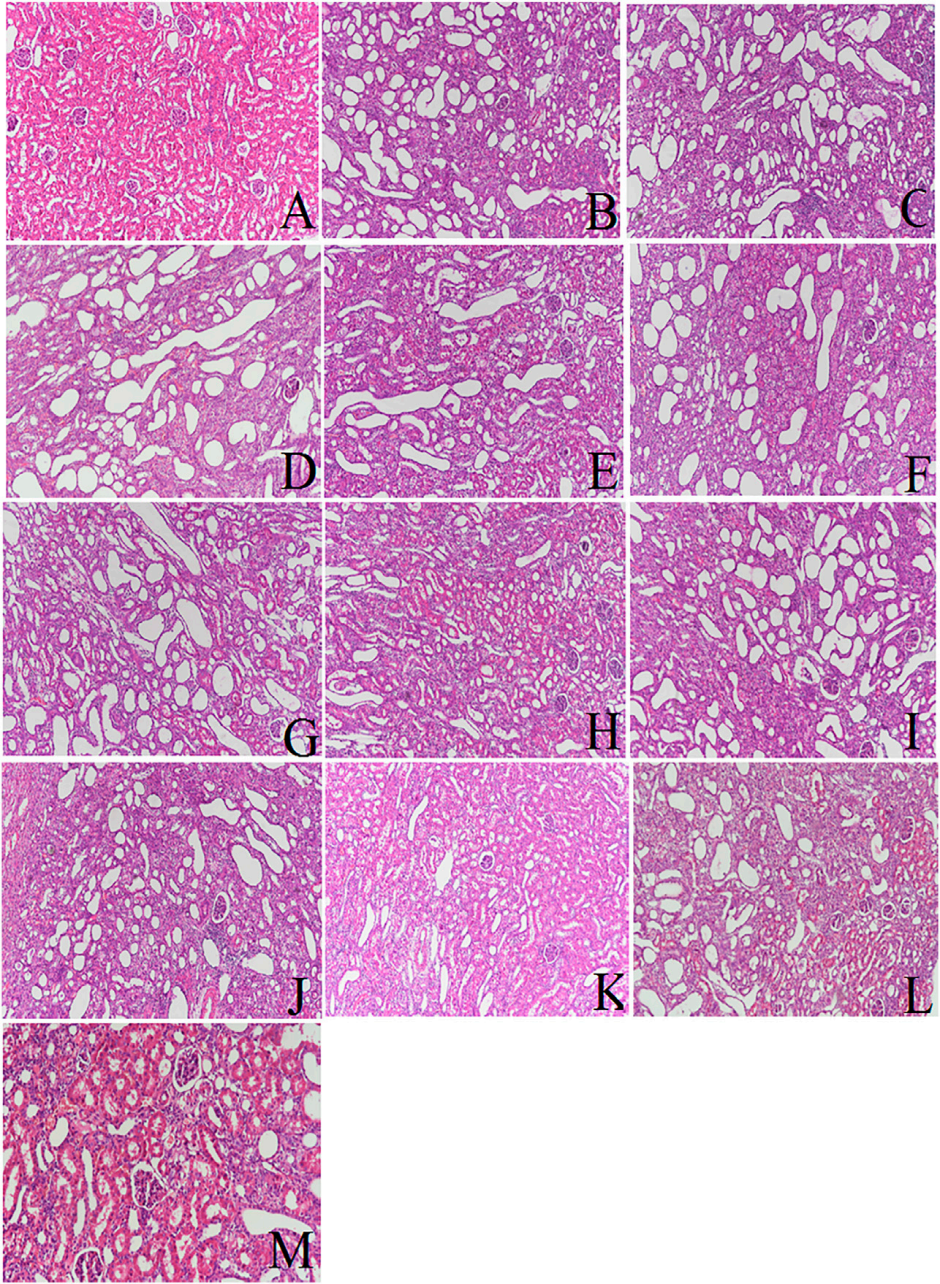
Renal pathological changes under different conditions (HE staining): **(A)**: sham; **(B)**: UUO on 7th day; **(C)**: UUO on 14th day; **(D)**: UUO on 21st day; **(E)**: rhein on 7th day; **(F)**: rhein on 14th day; **(G)**: rhein on 21st day; **(H)**: curcumin on 7th day; **(I)**: curcumin on 14th day; **(J)**: curcumin on 21st day; **(K)**: rhein plus curcumin on7th day; **(L)**: rhein plus curcumin on 14th day; **(M)**: rhein plus curcumin on 21st day.

**FIGURE 2:**
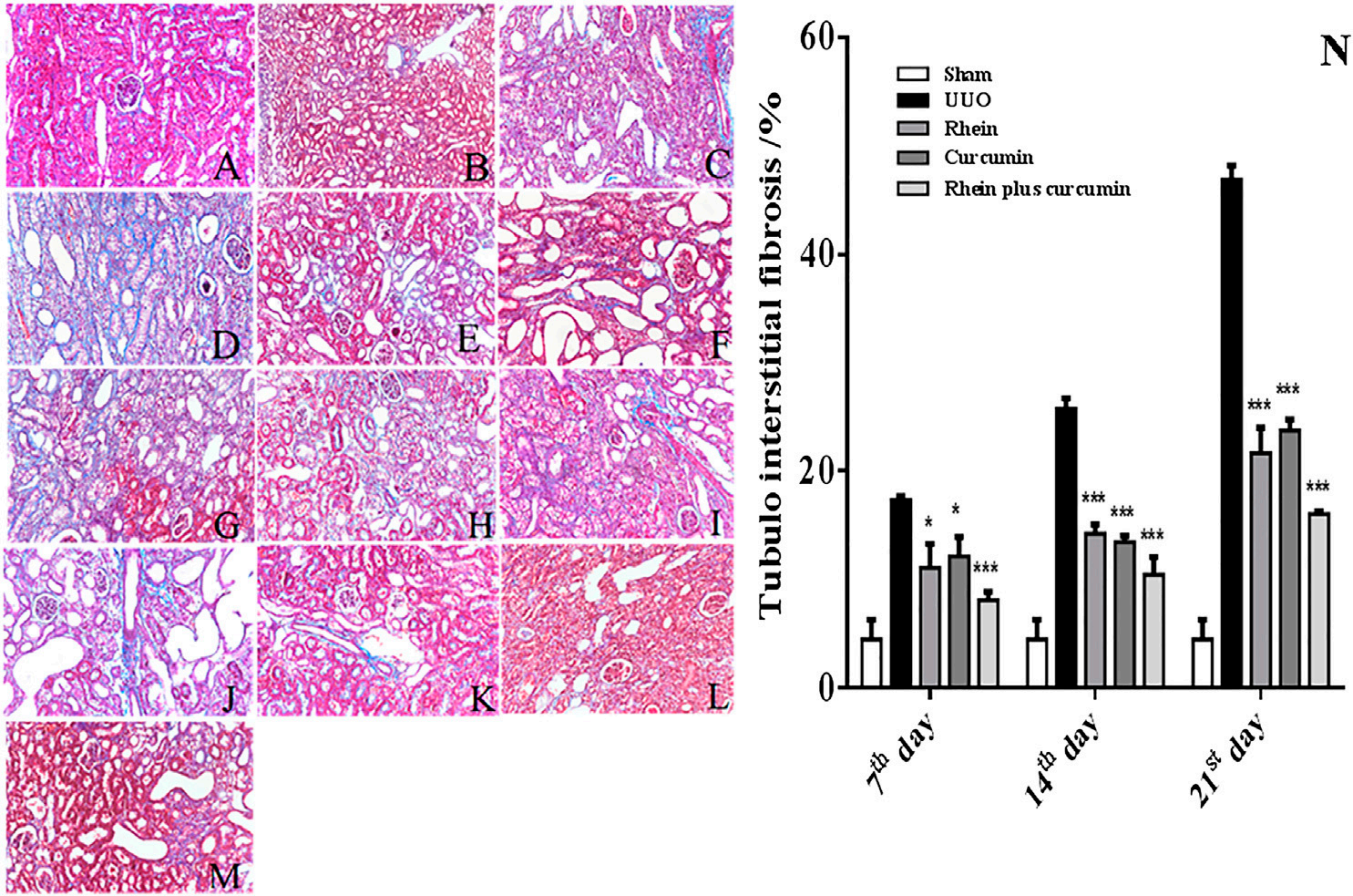
Renal pathological changes under different conditions (Masson staining): **(A)**: sham; **(B)**: UUO on 7th day; **(C)**: UUO on 14th day; **(D)**: UUO on 21st day; **(E)**: rhein on 7th day; **(F)**: rhein on 14th day; **(G)**: rhein on 21st day; **(H)**: curcumin on 7th day; **(I)**: curcumin on 14th day; **(J)**: curcumin on 21st day; **(K)**: rhein plus curcumin on 7th day; **(L)**: rhein plus curcumin on 14th day; **(M)**: rhein plus curcumin on 21st day. **(N)**: The degree of renal fibrosis in co-administration group and single rhein or curcumin group (*p < 0.05; **p < 0.01; ***p < 0.001).

The result of the HE and Masson staining is shown in Figure 1 and Figure 2, respectively. The morphology of renal sections in sham group was good: the structures of glomeruli and renal tubules were normal, and the tubules interstitial space was narrow. No inflammatory cell infiltration was observed. But in the UUO group, there was an expansion of the renal interstitial space, an increase in interstitial cells, a deposition of collagen components, the appearance of vacuoles, lesions of tubular and the fibrosis of renal interstitial was observed from the 7^th^ day after surgery operation. On the 14th day, in the UUO group, there was tubular atrophy, renal parenchyma was replaced by fibrous tissue, vacuoles were further increased, and collagen deposition and inflammatory cell infiltration were observed. On the 21st day, the UUO group had pathological changes of renal tissue that were more significant and the severity of the renal tissue injury was further exacerbated. Compared to the UUO groups, the treatment groups including rhein, curcumin and co-administration group had an obviously reduced degree of renal damage at the corresponding time points. The number of vacuoles were decreased. Collagen deposition and inflammatory cell infiltration was improved significantly and the degree of renal fibrosis was alleviated. In addition, in coadministration group, the degree of renal fibrosis was improved more than that of rhein or curcumin groups, which suggested that the combination of rhein and curcumin could treat renal interstitial fibrosis and improve the degree of renal tissue damage more effectively. As shown in Figure 2N, the degree of renal fibrosis was reduced obviously in the treatment groups, including rhein, curcumin and rhein plus curcumin groups at the corresponding time points, consistent with the result of HE and Masson staining.

#### The Levels of Serum Scr and BUN

The content of Scr and BUN in different groups was shown in Table 1. The average level of Scr and BUN was within the normal range in the sham-operated group. In UUO group, the level of Scr and BUN increased with time and was higher than that of the sham-operated group. In the 21st day UUO group, the level of Scr and BUN was 62.50 ± 11.11 and, 11.67 ± 2.50, respectively, which were significantly higher than those in the 7^th^ day UUO group (55.96 ± 7.24 and 9.10 ± 0.09, respectively). Compared to the UUO model group, the level of BUN was decreased in rhein, curcumin, and rhein plus curcumin treatment groups at the corresponding days. Moreover, the level of BUN in co-administration group was lower than those in the single-adminstration rhein or curcumin treatment group. The level of Scr in treatment groups and UUO group was significantly higher than sham-operated group (*p* < 0.05). Compared to the UUO group at the corresponding time points, the level of Scr in administrated groups was slightly lower, but was not significant.

**TABLE 1:** The levels of serum Scr and BUN.

Group	Scr	BUN
Sham	15.16 ± 2.93	5.62 ± 0.32
UUO 7th	55.96 ± 7.24^a^	9.10 ± 0.09^a^
UUO 14th	62.37 ± 6.35^a^	11.21 ± 0.76^a^
UUO 21st	62.50 ± 11.11^a^	11.67 ± 0.38^a^
UUO + rhein 7th	42.53 ± 5.01^a^	6.16 ± 0.12^a^
UUO + rhein 14th	55.74 ± 5.26^a^	8.14 ± 0.46^a, b^
UUO + rhein 21st	57.68 ± 6.41^a^	7.36 ± 0.29^a, b^
UUO + curcumin 7th	55.01 ± 4.7^a^	9.35 ± 1.06^a, b^
UUO + curcumin 14th	59.87 ± 7.30^a^	10.58 ± 0.83^a^
UUO + curcumin 21st	61.41 ± 6.26^a^	10.81 ± 1.70^a^
UUO + coadministration 7th	62.87 ± 8.90^a^	7.52 ± 0.32^a, b^
UUO + coadministration 14th	52.99 ± 9.79^a^	8.03 ± 0.44^a, b^
UUO + coadministration 21st	53.27 ± 5.26^a^	7.43 ± 0.07^a, b^

a The comparison between sham-operated group and other groups.

b The comparison between treatment groups and UUO at the corresponding days.

### Method Validation

No obvious endogenous interference at the retention time of IS and rhein or curcumin (Supplementary Figure S2) indicated that the selectivity of the established method were satisfactory. As shown in Supplementary Table S1 and Supplementary Table S2, the matrix effects and recovery rate of rhein and curcumin in plasma and tissue were in the range of 90.3%–96.3% and 81.9%–89.3%, respectively. The calibration curve showed good linearity in the range of 1–2000 ng/ml for rhein, 1–200 ng/ml for curcumin. The lower limit of quantification (LLOQ) of both rhein and curcumin was determined at 1 ng/ml. Supplementary Table S3 showed that the intra-/inter-day precisions (RSD) of rhein and curcumin was less than 4.8% and 5.1%, respectively. The intra-/inter-day accuracies of rhein and curcumin were ranged from 81.7% to 93.5% and from 83.4% to 93.1%, respectively. As shown in Supplementary Table S4 and Supplementary Table S5, the stability of rhein and curcumin storage in different conditions was less than 7.3%. All detailed information was described in supporting information.

### Pharmacokinetics Study

**FIGURE 3:**
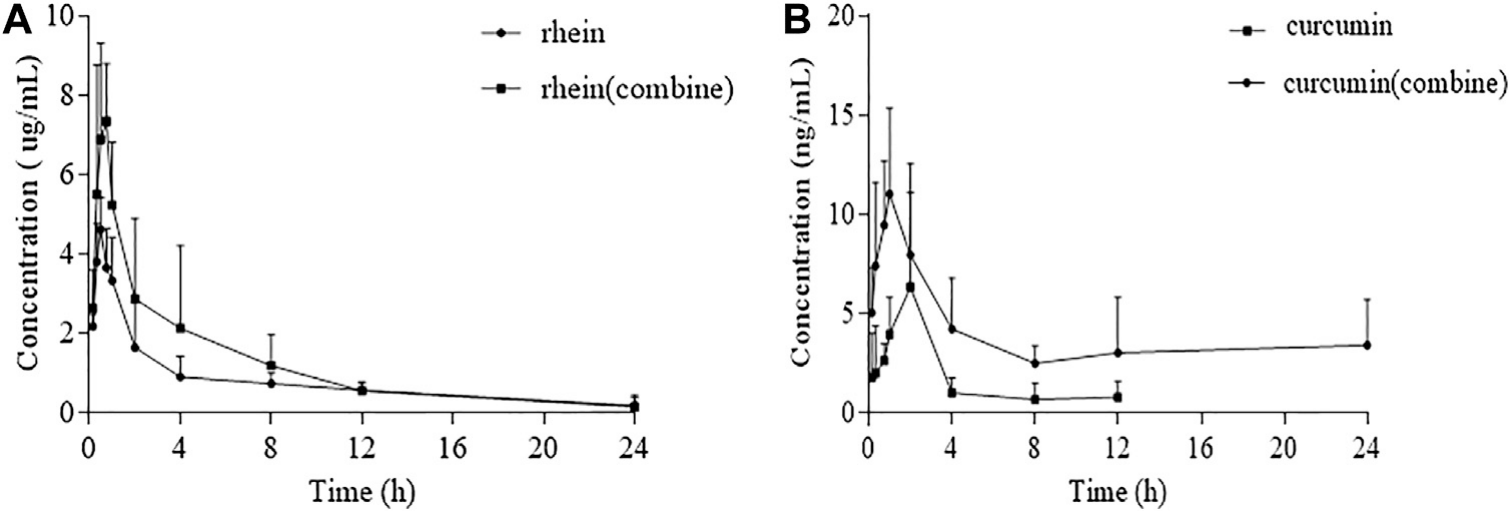
Plasma concentration-time profile: **(A)**: Plasma concentration-time changes of rhein administrated alone and in combination in rats after intragastric administrations (n = 6); **(B)**: Plasma concentration-time changes of curcumin administrated alone and in combination in rats after intragastric administrations (n = 6).

The established UPLC-MS/MS method was successfully applied to pharmacokinetic and renal tissue distribution studies of rhein and curcumin in biological samples. Figure 3 showed the mean plasma concentration-time profiles for rhein, curcumin and their combination. In general, rhein and curcumin had a higher concentration in plasma after co-administration. The corresponding pharmacokinetic parameters of rhein fitted by non-compartmental model were represented in Table 2. For single administration, the apparent volume of distribution (V_d_) was 44.21 ± 27.16 L/kg. The apparent volume of distribution (V_d_) of rhein after co-administration was 27.96 ± 18.78 L/kg, indicating that the rhein concentration in rat plasma was elevated. The AUC and C_max_ of rhein in co-administration was 27,497.71 ± 12,297.14 μg/L*h and 8,254.74 ± 2,438.31 μg/L, respectively, which was significantly higher than that of single-administration (AUC = 18,964.28 ± 2,261.96 μg/L*h, C_max_ = 4,815.48 ± 699.91 μg/L). However, the half-life (T_1/2_) of rhein was 6.83 ± 5.43 h in single-adminstration and 4.40 ± 1.80 h after co-administration, indicating that the elimination process of rhein *in vivo* was slightly affected by curcumin. Similarly, there was no evident difference between the clearance rate (Cl) for single administration (4.91 ± 0.94 L/h/kg) and co-administration (4.10 ± 1.51 L/h/kg).

**TABLE 2:** Changes on main pharmacokinetic parameters of rhein and curcumin in rat plasma after their combination (n = 6).

	Parameters	Unit	Rhein/Curcumin	Rhein + Curcumin
Rhein	AUC _(0-t)_	μg/L*h	18,964.3 ± 2,262.0	27,497.7 ± 12,297.1
	Cl	L/h/kg	4.91 ± 0.94	4.1 ± 1.5
	V_d_	L/kg	44.2 ± 27.2	28.0 ± 18.8
	C_max_	μg/L	4,815.5 ± 699.9	8,254.7 ± 2,438.3
	T_1/2_	H	6.9 ± 5.4	4.4 ± 1.8
	T_max_	H	0.49 ± 0.15	0.60 ± 0.18
Curcumin	AUC _(0-t)_	μg/L*h	19.2 ± 10.8	88.0 ± 28.7
	Cl	L/h/kg	4,694.5 ± 2,977.3	1,015.2 ± 557.7
	V_d_	L/kg	31,715.2 ± 24,232.9	9,520.0 ± 7,430.4
	C_max_	μg/L	6.6 ± 4.2	27.7 ± 18.7
	T_1/2_	H	6.7 ± 5.7	10.9 ± 14.3
	T_max_	H	1.4 ± 0.9	1.1 ± 0.7

AUC, Area under the curve; Cl, Clearance; C_max_, Maximal plasma concentration; T_1/2_, Elimination half–life; T_max_, Time to reach maximal plasma concentration; V_d_, Volume of distribution.

As shown in Table 2, the apparent volume of distribution (V_d_) of curcumin was 31,715.21 ± 24,232.87 L/kg and reduced to 9,519.99 ± 7,430.36 L/kg after co-administration. Although the V_d_ decreased approximately 3-fold after co-administration, curcumin was mostly distributed in specific tissues or organs. The AUC and C_max_ of curcumin were significantly increased from 19.19 ± 10.84 μg/L*h to 87.98 ± 28.73 μg/L*h and from 6.56 ± 4.18 μg/L to 27.67 ± 18.68 μg/L for C_max_, respectively. This was an approximately 4-fold increase in AUC and C_max_ after co-administration, which showed there was an increase in the absorption of curcumin. In the contrary, the clearance rate (Cl) was reduced by about 75%, indicating that the elimination of curcumin slowed down after co-administration.

**FIGURE 4:**
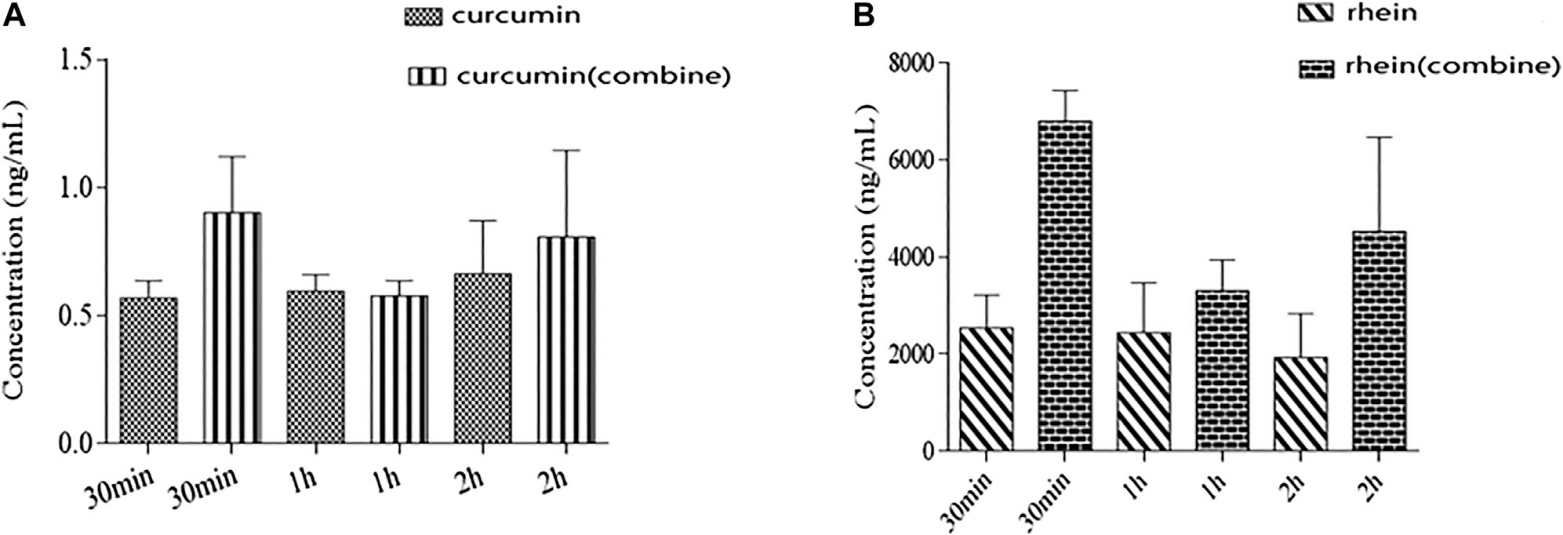
Tissue concentration profile: **(A)**: rhein alone and in combination with curcumin in renal tissue after co-administration 30 min, 1 and 2 h; **(B)**: curcumin alone and in combination with rhein in renal tissue after co-administration 30 min, 1 and 2 h.

We also detected the content of rhein and curcumin in renal tissue after oral administration. The results are summarized in Figure 4. As shown in Figure 4, co-administration contributed to higher levels of rhein and curcumin in renal tissue, especially at 30 min post-administration. It has been proved that rhein is mainly distributed in the liver, spleen, kidney, heart, lung and other tissues with rich blood supply after oral administration, suggesting that the distribution of rhein depends on the blood flow or perfusion rate of organs. The distribution of rhein in the liver is the most, followed by that in the spleen and kidney (Sun et al., 2012). The concentration of rhein in renal tissue was increased more than 2-fold after co-administration (2.62 ± 0.70 μg/ml vs. 7.00 ± 0.65 μg/ml), indicating that combination with curcumin promoted the affinity of rhein with renal tissue. A previous study on the tissue distribution of curcumin showed that the concentration of curcumin in the heart, liver and kidney was extremely low (Gutierres et al., 2015; Arozal et al., 2019). In this study, the concentration of curcumin in renal tissue was determined at three different periods including 30 min, 1 h, 2 h after administration. The measured concentration of curcumin in renal tissue was very low when administrated on its own, which was consistent with the literatures (Gutierres et al., 2015; Arozal et al., 2019). However, after combination with rhein, the level of curcumin in renal tissue was nearly doubled in 30 min after administration. In brief, the distribution of rhein and curcumin in renal tissue was increased significantly after co-administration, which was quite different from that in single-administration.

### Docking Studies

**FIGURE 5:**
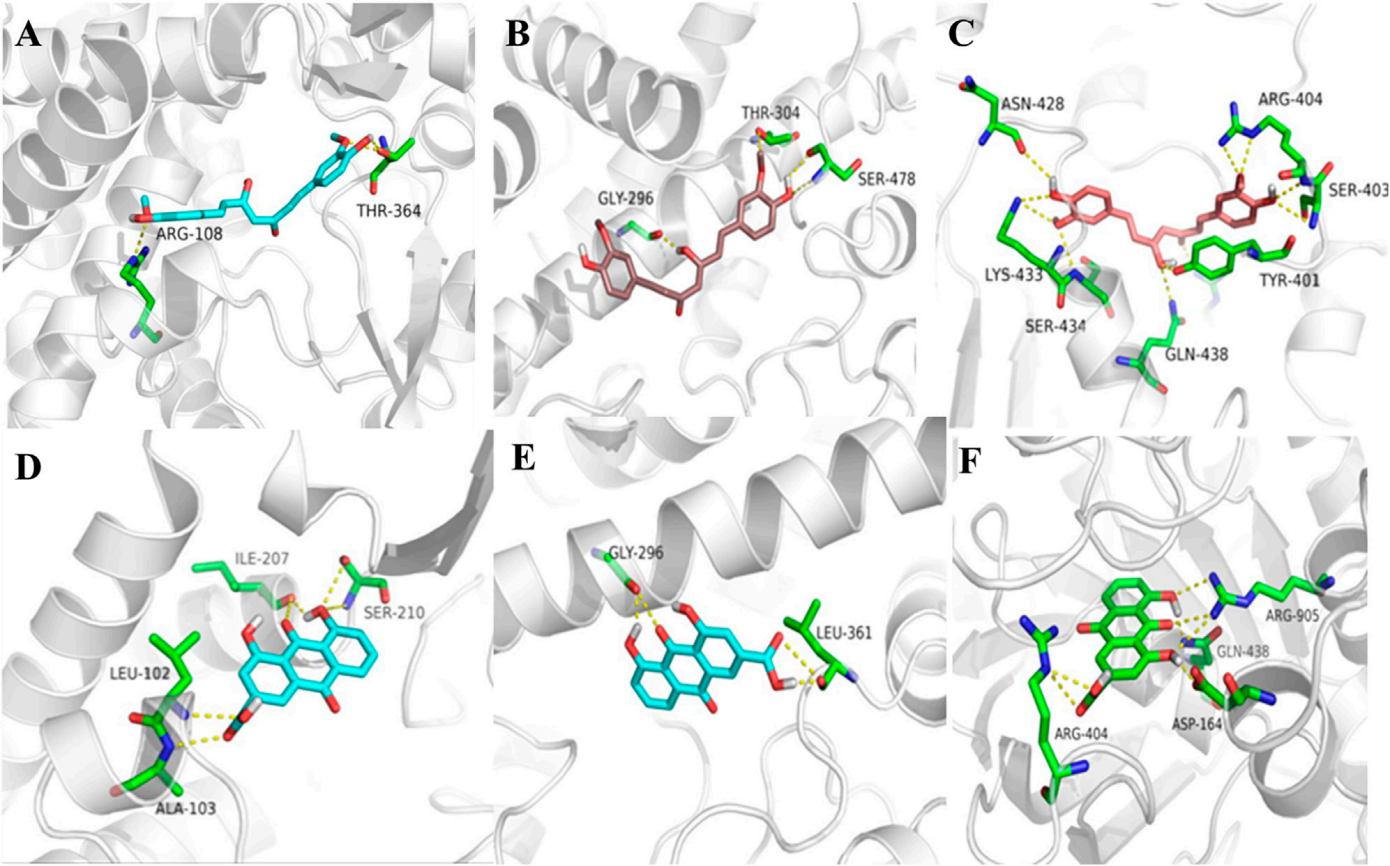
3D docking conformation of rhein and curcumin: **(A)**: 3D docking conformation of curcumin with CYP2C9; **(B)**: 3D docking conformation of curcumin with CYP2C19; **(C)**: 3D docking conformation of curcumin with P-gp; **(D)**: 3D docking conformation of rhein with CYP2C9; **(E)**: 3D docking conformation of rhein with CYP2C19; **(F)**: 3D docking conformation of rhein with P-gp.

In order to explore the interaction of rhein and curcumin, they are docked with P-gp, CYP2C9, and CYP2C19, respectively. The results of docking studies are shown in Figure 5. The results showed that both rhein and curcumin could bind to P-gp, CYP2C9, and CYP2C19. The binding energy for rhein to P-gp, CYP2C9, and CYP2C19 was −9.04, −7.40 and −6.23 kcal/mol, respectively, while the binding energy for curcumin to P-gp, CYP2C9, and CYP2C19 was 9.56, −7.84, −8.44 kcal/mol, respectively. All the values were less than -5 kcal/mol, indicating that two compounds could interact with P-gp and CYP2C19, consistent with the results reported in the literatures (Tang et al., 2009; Bahramsoltani et al., 2017). Moreover, both rhein and curcumin were bound to the GLY-296 binding sites of CYP2C19, as well as GLN-438 and ARG-404 binding sites of P-gp. Therefore, we speculated that there was interation between curcumin and rhein *in vivo*.

### Interaction Between Rhein and Curcumin *in vitro*

**FIGURE 6:**
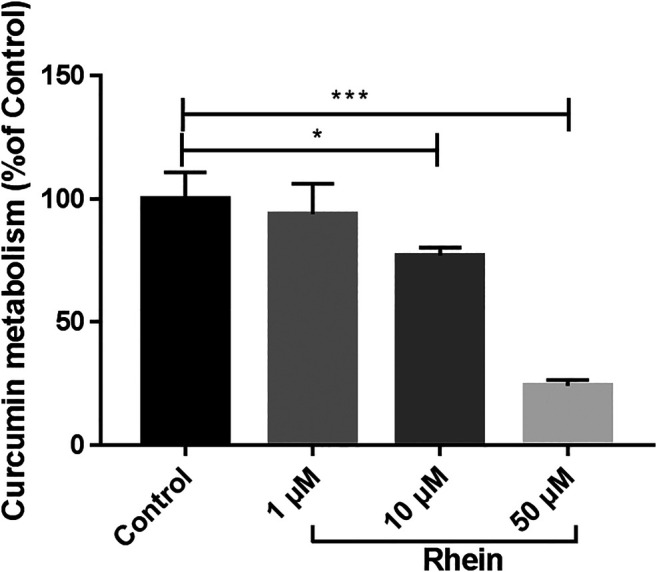
Relatively metabolic amount of curcumin after incubation with different concentrations of rhein. *p < 0.05, ***p < 0.01, compared to control group.

The metabolic interaction between rhein and curcumin through CYP2C9 was shown in Figure 6. Rhein exhibited the potential inhibition against curcumin by reducing the metabolism of curcumin. As shown in Figure 6, when a lower concentration of rhein (10 μM) was used, the metabolism of curcumin was inhibited by 22.9% (*p* = 0.026). When a higher concentration of rhein (50 μM) was used, the metabolism of curcumin was inhibited by 75.8% (*p* = 0.0001). Thus, rhein had an inhibitory effect on the metabolism of curcumin by inhibiting CYP2C9.

## Discussion

Inflammation factor including TGF-β, MCP-1, IL-1β, and IL-6 colud promote the occurrence and progression of CKD. Rhein and curcumin, the effective ingredients of BSHX, demonstrated anti-inflammatory effect. It has reported that rhein could exert anti-inflammatory and antifibrotic effects through down-regulation NF-κB related pathway and inhibition of TGF-β/Smad3 pathway (Guan et al., 2015). Similarly, the anti-inflammatory and antifibrotic effect of curcumin has also been confirmed. The curcumin properties on anti-inflammatory were mediated via suppressing the key inflammatory factors such as cyclooxygenase-2, 5-lipoxygenase and inducible nitric oxide synthase (Jurenka, 2009; Mirzaei et al., 2017). However, because of the low oral bioavailability of curcumin, its widespread use in the treatment of CKD has been limited for decades (Anand et al., 2007; Mirzaei et al., 2017). The goal of this study was to investigate whether there is a synergistic antifibosis effect between rhein and curcumin when administrated to rat in combination, and to provide an interaction mechanism between rhein and curcumin.

The renal pathological changes including interstitial fibrosis and tubular atrophy were obviously improved by co-administrated rhein and curcumin, the BUN level had reduced significantly as well, suggesting that they had a synergistic effect on treatment of CKD. In addition, as evidenced by the plasma concentration-time profiles, the AUC and C_max_ of rhein and curcumin was significantly increased after coadministration, but the Cl was reduced, indicating that the exposure of rhein and curcumin in plasma was higher, and their elimination was slowed down. We also compared the concentration of rhein, curcumin and their combination in renal tissue after oral administration. The higher level of rhein and curcumin in renal tissue after their combination was observed, especially at 30 min after administration. Meanwhile, we found that the clearance of curcumin was significantly reduced after the co-administration, with an approximately 25% reduction. Such changes not only improved the bioavailability of the rhein and curcumin, but also suggested that there was a synergistic anti-fibrosis effect between two compounds.

A majority of reported clinically relevant drug-drug interaction mechanism include CYPs inhibition. According to the previous studies, the major metabolic enzyme of rhein is CYP2C19 (He et al., 2015). Meanwhile, curcumin inhibits CYP2C9 and CYP2C19, while rhein inhibis CYP2C9 (Tang et al., 2009; Bahramsoltani et al., 2017), which may be the main reason for the reduced elimination rate, leading to the increase in AUC and C_max_ of rhein. Molecular docking studies on CYP2C19 inhibition revealed that inhibition by rhein and curcumin occurred via GLY-296. The previous studies showed that rhein could activate the efflux transport mediated by P-gp (Yang et al., 2017), while curcumin could inhibit P-gp (Ampasavate et al., 2010). Thus, rhein and curcumin have opposite effects on P-gp activity.

## Conclusion

In this study, the pharmacokinetics and pharmacodynamics of rhein and curcumin by single and combined administration were explored. Compared with single component administration, co-administration of rhein and curcumin significantly increasesd their concentration in plasma and tissue, showing better efficacy in delaying the progression of CKD. The increased level in plasma and renal tissue distribution through inhibiting metabolic enzymes may be the potential mechanism for this. In conclusion, this study demonstrated that there was a synergistic antifibrosis effect of rhein and curcumin in ameliorateing chonic kidney disease, and provided an important explanation on the mechanism of their synergy from a pharmacokinetic viewpoint.

## Data Availability Statement

The raw data supporting the conclusions of this article will be made available by the authors, without undue reservation, to any qualified researcher.

## Ethics Statement

The animal study was reviewed and approved by Animal Ethics Committee in Wenzhou Medical University. Written informed consent was obtained from the owners for the participation of their animals in this study.

## Author Contributions

XH, YC and GL accomplished all the animal experiments, analyzed, interpreted the data, and drafted the manuscript. QX and XY helped to develop the method for LC–MS/MS measurement and analyzed the data. XL and XY conducted the animal experiments and revised the manuscript. ZX contributed to study design, data analysis, and revising the manuscript. All authors have read and approved the final version of the manuscript.

## Funding

This work was supported by Wenzhou Science and Technology Major Project, China (ZS2017018), National Natural Science Foundation of China (No. 81773691 and 81973696) and 13th Five -Year plan of Zhejiang Province Traditional Chinese Medicine (Integrated Chinese and Western Medicine) Key Discipline Construction (Integrated Traditional Chinese and Western Medicine Pharmacy 2017-XK-A37).

## Conflict of Interest

The authors declare that the research was conducted in the absence of any commercial or financial relationships that could be construed as a potential conflict of interest.
