# Supplementary material for: Pharmacokinetics and Pharmacodynamics of the Combination of Rhein and Curcumin in the Treatment of Chronic Kidney Disease in Rats
Source: Front Pharmacol. 2020 Dec 23;11:573118. doi: 10.3389/fphar.2020.573118 (PMC7785804; doi:10.3389/fphar.2020.573118)
Supplement: Supplementary file 1 [file table1.docx]

Supplementary Material

**Pharmacokinetics and pharmacodynamics of the combination of rhein and curcumin in the treatment of chronic kidney disease in rats**

Xiaoying He^1^, Guowei Li^1^, Yuanyuan Chen^1^, Zheng Xiang^1^*, Chenglv Hong^2^*

^1^ School of Pharmaceutical Sciences, Wenzhou Medical University, Wenzhou, 325035, China

^2^ Department of Cardiology, The First Affiliated Hospital of Wenzhou Medical University, Wenzhou, Zhejiang, China

*Corresponding author:

Chenglv Hong

clhong2019@sina.com

Zheng Xiang

XZH0077@126.com

Xiaoying He, [1018625666@qq.com](mailto:1018625666@qq.com); Xiaojun Cai, [xiaocaixj@163.com](mailto:xiaocaixj@163.com)

Guowei Li, [1976447084@qq.com](mailto:1976447084@qq.com); Yuanyuan Chen, eastinnocyy@163.com

**Method**

**Liquid chromatography - tandem mass spectrometry (LC–MS/MS) analysis**

The separation of rhein and curcumin was achieved by a Waters Acquity-UPLC system, which connected to Waters XEVO TQD triple-quadrupole MS (Waters Corp., Milford, MA, USA). 0.1% formic acid in water (A) and acetonitrile (B) was selected as the mobile phase. Gradient elution program was performed as follows: 0-0.2 min, B (10%); 0.2-1.5 min, B (10%-80%)；1.5-2 min, B (80%)；2-2.2 min, B (80%-10%)；2.2-4 min, B (10%)；During the analysis period, the temperature of the column was set at 35 ℃ and the flow rate was 0.3 mL/min.

The optimal mass spectrometric parameters were chosen as follows: capillary voltage, 1.5 kV; sample cone, 50 V; source temperature, 100 °C; desolvation temperature 400 °C; cone gas flow rate 50 L/h; desolvation gas (N2) flow rate 800 L/h. Electrospray ion source (ESI) and multiple reaction monitoring (MRM) mode were used for mass detections. The collision energy of rhein, curcumin and IS was all 30 eV. Detection of rhein and IS was conducted in negative mode with a range of the m/z 283.2→182.89, m/z 611.30→302.27, respectively. In the meantime, the detection of curcumin was conducted in positive mode with a range of the m/z 369.2→177.05.

**Preparation of stock solution and quality control (QC) samples**

The stock solution of rhein, curcumin and IS were prepared in methanol at a concentration of 120 µg/mL, 600 µg/mL, 300 µg/mL, respectively. The working standard solutions of rhein and curcumin at a series of concentration were obtained by precise dilution with methanol. All obtained solutions were stored at 4 °C until analysis. The concentration at 1 ng/mL, 50 ng/mL, 1600 ng/mL of rhein and 2 ng/mL, 50 ng/mL, 160 ng/mL of curcumin were used as quality control (QC) samples.

**Method validation**

According to the latest Food and Drug Administration (FDA) guidelines, the method validation was accomplished in term of selectivity, linearity, precision, accuracy, matrix effect, recovery rate and stability to prove the reliability and reproducibility of the established method.

**Liquid chromatography - tandem mass spectrometry (LC–MS/MS) analysis**

In order to obtain the optimal mass spectrum conditions, the detection of rhein and IS was conducted in negative mode, while curcumin was detected in positive mode. Under the optimized conditions with a gradient elution program, the retention time of rhein, curcumin and IS was 2.17, 2.22, 1.74 min, respectively. The MS/MS spectrogram of rhein, curcumin and IS were shown in Supporting Fig. S1.

**Selectivity**

There was no obvious endogenous interference at the retention time of IS and rhein or curcumin (Fig.S2), indicating that the selectivity of the established method were satisfactory.

**Recovery and matrix effect**

The matrix effects of rhein and curcumin in plasma were 93.8 % - 96.3 %, 92.7 % - 96.3 %, respectively, which was evaluated at three levels concentration. Similarly, the matrix effects of rhein and curcumin in renal tissue were 90.3 % - 94.1 %, 90.7 % - 93.7 %, respectively. For IS, the matrix effect in plasma and renal tissue was 92.7 % ± 0.5 %, 90.5% ± 3.1%, respectively. The recovery rate of rhein and curcumin was also assessed, ranged from 81.9 % to 88.6 %, 82.4 % to 89.3 % in plasma, ranged from 82.5 % to 84.9 %, 82.6 % to 85.0 % in renal tissue, respectively. The recovery rate of IS was 89.2 % ± 4.3 % in plasma and 85.4 % ± 4.3 % in renal tissue. All results of matrix effects and recoveries were summmarized in Table S1and Table S2.

**Calibration curve, Precision and accuracy**

The mean linear regression equation of rhein and curcumin was *y=0.0026x+0.0333, y=0.2622x-0.0952*, respectively. The calibration curve showed good linearity in the range of 0.5-2000 ng/mL for rhein with the correlation coefficient r ≥ 0.9903, and 1-200 ng/mL for curcumin with the correlation coefficient r ≥ 0.9988. The lower limit of quantification (LLOQ) of rhein and curcumin was determined at 1 ng/mL and 2 ng/mL, respectively. In term of precision and accuracy, the intra-/inter-day precisions (RSD) of rhein was less than 4.5 % and 5.1 %, respectively. The intra-/inter-day accuracies of rhein were ranged from 81.7 % to 91.8 % and from 84.6 % to 93.5 %, respectively. For curcumin, the intra-/inter-day precisions (RSD) was less than 4.8 % and 3.9%, respectively. The intra-/inter-day accuracies of rhein were ranged from 84.2 % to 93.1% and from 83.4% to 92.8%, respectively. Both precision and accuracy of rhein and curcumin was in accordance with FDA guideline, and all variation was within ± 20 %. The reults were listed in Table S3.

**Stability**

The stability (RSD) of rhein and curcumin storage at room temperature for 12 h, 4°C for 24 h, and -20 °C for 20 days was less than 7.3 %. The stability (RSD) of rhein and curcumin after three freeze (−80 °C) - thaw (RT) cycles was less than 3.2 % and 4.3 %, respectively. The analysis results were summarized in Table S4 and Table S5. All the results indicated that rhein and curcumin was stable during the analysis period.

**Figure legends**

**Fig.S1** MS/MS spectra A: rhein; B: curcumin; C:IS.

**Fig.S2** The representative MRM chromatograms of rhein, curcumin and IS; A: blank rat plasma; B: blank rat plasma spiked with rhein, curcumin and IS; C: rat plasma after intragastric administration.

**Fig. S1**

**Fig. S2**


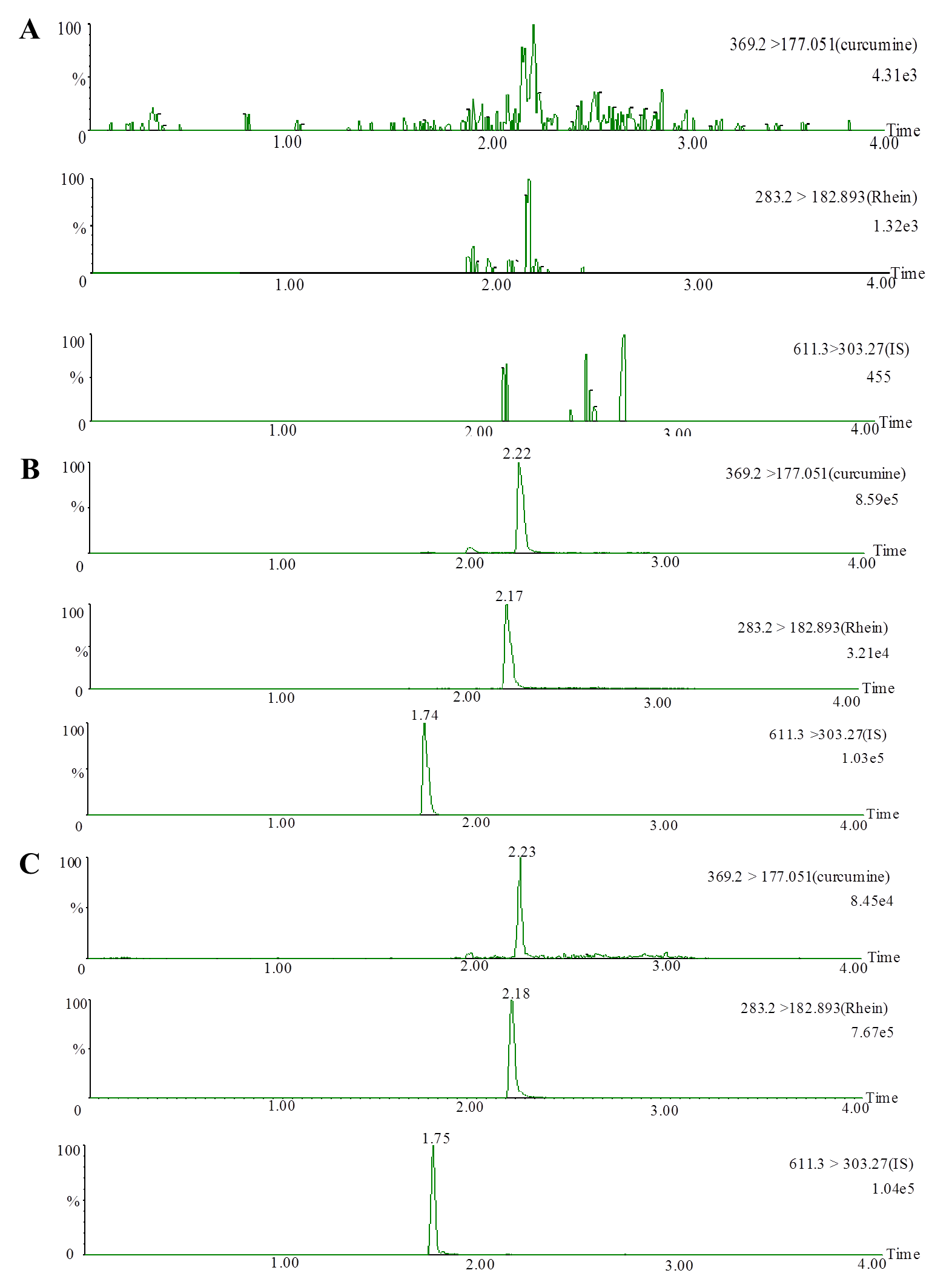


**Table S1 Matrix effect and extraction recovery of rhein, curcumin and IS in plasma (n=6)**

|  | Concentration (ng/mL) | Extraction recovery (%) | Matrix effect (%) |
| --- | --- | --- | --- |
| rhein | 1 | 81.9±0.3 | 93.8±0.2 |
|  | 50 | 84.2±0.0 | 96.3±1.5 |
|  | 1600 | 88.6±9.1 | 95.6±6.2 |
| curcumin | 2 | 82.4±0.4 | 92.7±0.2 |
|  | 50 | 89.3±0.1 | 94.8±0.3 |
|  | 160 | 86.3±3.1 | 96.3±5.7 |
| IS | 200 | 89.2±4.3 | 92.7±0.5 |

**Table S2 Matrix effect and extraction recovery of rhein, curcumin and IS in renal tissue (n=6)**

|  | Concentration (ng/mL) | Extraction recovery (%) | Matrix effect (%) |
| --- | --- | --- | --- |
| rhein | 1 | 84.9±2.2 | 92.4±1.5 |
|  | 50 | 82.5±4.9 | 94.1±1.7 |
|  | 1600 | 84.0±5.3 | 90.3±5.2 |
| curcumin | 2 | 82.6±2.9 | 90.7±1.5 |
|  | 50 | 85.0±1.7 | 91.6±1.3 |
|  | 160 | 82.8±4.8 | 93.7±4.7 |
| IS | 200 | 85.4±4.3 | 90.5±3.1 |

**Table S3 Precision and accuracy of rhein and curcumin (n=6)**

| Name | Concentration (ng/mL) | | | Precision RSD (%) | Accuracy (%) |
| --- | --- | --- | --- | --- | --- |
|  | Spiked (ng/mL) | | Found (mean ± SD) (ng/mL) |  |  |
| rhein | *Intra-day* |  | |  |  |
|  | 1 | 0.82 ± 0.04 | | 4.5 | 81.7 |
|  | 50 | 43.13 ± 1.30 | | 3.0 | 86.3 |
|  | 1600 | 1467.21 ± 20.39 | | 1.4 | 91.8 |
|  | *Inter-day* |  | |  |  |
|  | 1 | 0.85 ± 0.04 | | 5.1 | 84.6 |
|  | 50 | 46.33 ± 1.74 | | 3.7 | 92.7 |
|  | 1600 | 1496.01 ± 29.82 | | 2.0 | 93.5 |
| curcumin | *Intra-day* |  | |  |  |
|  | 2 | 1.84 ± 0.04 | | 4.8 | 84.2 |
|  | 50 | 46.53 ± 1.96 | | 4.2 | 93.1 |
|  | 160 | 148.21 ± 2.23 | | 1.5 | 92.6 |
|  | *Inter-day* |  | |  |  |
|  | 2 | 1.83 ± 0.03 | | 3.9 | 83.4 |
|  | 50 | 46.38 ± 1.46 | | 3.2 | 92.8 |
|  | 160 | 146.21 ± 3.13 | | 1.6 | 91.4 |

**Table S4 Stability of rhein (n=3)**

| Spiked (ng/mL) | Condition | Found (mean ± SD) (ng/mL) | RSD (%) |
| --- | --- | --- | --- |
| 1 | Room temperature for 12 h | 0.82 ± 0.05 | 6.2 |
|  | 4℃ for 24 h | 0.74 ± 0.05 | 7.2 |
|  | -20℃ for 20 days | 0.76 ± 0.07 | 4.8 |
|  | Three freeze-thaw cycles | 0.75 ± 0.02 | 3.2 |
| 50 | Room temperature for 12 h | 43.89 ± 2.60 | 5.9 |
|  | 4℃ for 24 h | 46.87 ± 2.83 | 6.0 |
|  | -20℃ for 20 days | 41.25 ± 1.54 | 3.7 |
|  | Three freeze-thaw cycles | 43.77 ± 0.99 | 2.3 |
| 1600 | Room temperature for 12 h | 1535.88 ± 28.94 | 1.9 |
|  | 4℃ for 24 h | 1543.81 ± 38.81 | 2.5 |
|  | -20℃ for 20 days | 1543.55 ± 33.48 | 2.2 |
|  | Three freeze-thaw cycles | 1533.66 ± 38.14 | 2.5 |

**Table S5 Stability of curcumin (n=3)**

| Spiked (ng/mL) | Condition | Found (mean ± SD) (ng/mL) | RSD (%) |
| --- | --- | --- | --- |
| 2 | Room temperature for 12 h | 1.89 ± 0.06 | 6.9 |
|  | 4℃ for 24 h | 1.92 ± 0.03 | 3.6 |
|  | -20℃ for 20 days | 1.66 ± 0.05 | 7.3 |
|  | Three freeze-thaw cycles | 1.92 ± 0.04 | 4.3 |
| 50 | Room temperature for 12 h | 46.89 ± 2.58 | 5.5 |
|  | 4℃ for 24 h | 48.27 ± 2.71 | 2.7 |
|  | -20℃ for 20 days | 41.59 ± 1.53 | 3.7 |
|  | Three freeze-thaw cycles | 47.43 ± 1.70 | 3.6 |
| 160 | Room temperature for 12 h | 152.55 ± 3.07 | 2.0 |
|  | 4℃ for 24 h | 155.28 ± 4.24 | 2.7 |
|  | -20℃ for 20 days | 146.89 ± 2.95 | 2.0 |
|  | Three freeze-thaw cycles | 153.40 ± 4.03 | 2.6 |
